# Multiple Acquired Elastotic Hemangioma in a Single Patient: A Case Report

**DOI:** 10.31729/jnma.5186

**Published:** 2020-10-31

**Authors:** Pramisha Kharel, Jia Chen, Pukar Chapagain, Himal Panth

**Affiliations:** 1 Department of Dermatology, Shanghai Dermatology Hospital, Shanghai, China; 2 Department of Dermatology, BP Koirala Institute of Health Sciences, Dharan, Nepal; 3 Universal College of Medical Sciences, Bhairahawa, Nepal

**Keywords:** *acquired elastotic hemangioma*, *basal cell carcinoma*, *elastosis*

## Abstract

Acquired elastotic hemangioma is a hemangioma variant which was first described in 2002. Usually it is characterized by being a benign, solitary, slow growing lesion and associated with solar exposure but here we discuss a case of 50 years old male from Australia, who had multiple pigmented and violaceous lesions on the back which he had noticed for 5 years. The cases of multiple lesions are reported few in the literature. The lesions had characteristic clinical dermatoscopic feature showing typical violaceous lesions with widespread shiny white structure without any vessels. Histopathology revealed band like proliferation of blood vessels involving superficial dermis arranged horizontally parallel to the epidermisalong with elastosis in dermis. Treatment was done through surgical excision with no relapse reported.

## INTRODUCTION

Acquired elastotic hemangioma was first reported by Luis Requena along with Heinz Kutzner and Thomas Mentzel. They gave the original description of this new variant of cutaneous hemangioma in the September 2002 issue of the Journal of the American Academy of Dermatology. They had described about the clinical, pathological and immunohistochemistry of this lesion initially in six women.^1^ Since then it has only been reported in 28 additional patients.^2–5^ It is a slow growing,erythematous, well-defined plaque which is usually asymptomatic and develops on the sun exposed areas.^6^ The most common location is the forearm, upper-arm, head, neck, back and chest. Here, we report a case of a man who had multiple lesions on his back.

**Figure 1:**
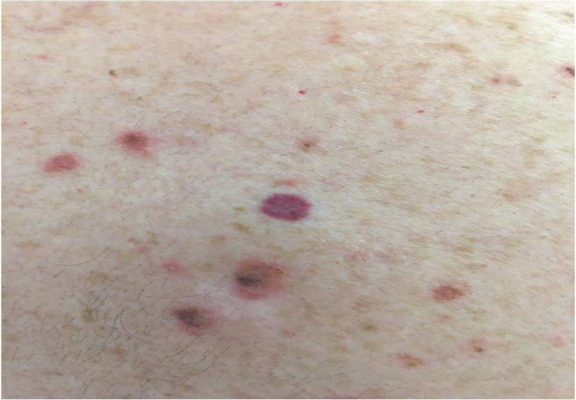
Lesion of acquired elastotic hemangioma.

**Figure 2:**
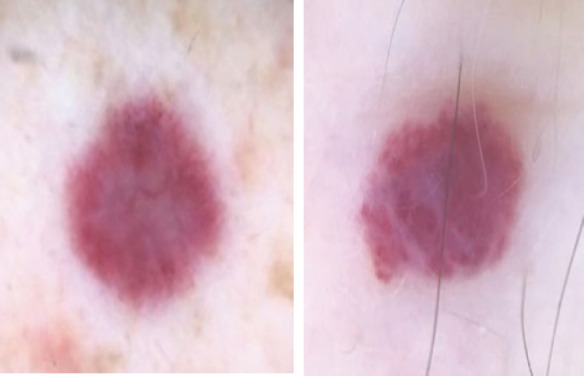
Dermatoscopy of AEH showing violaceous lesion with widespread shiny white structures without vessels.

**Figure 3:**
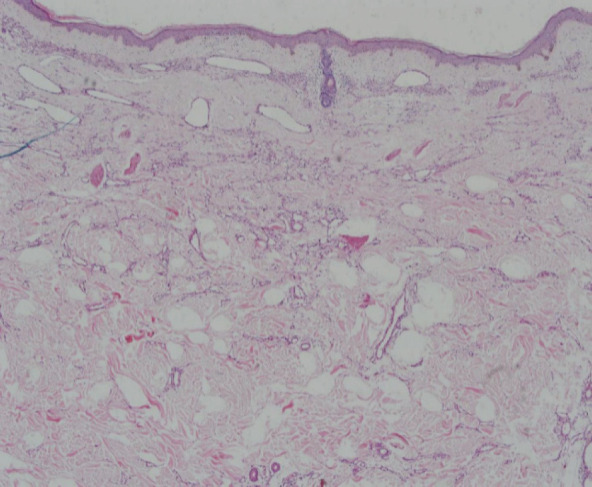
Band like proliferation of capillary blood vessels involving superficial dermis and arranged horizontally parallel to the epidermis along with elastosis in the dermis (Hematoxylin-eosin stain).

## CASE REPORT

A 50-year-old man presented with asymptomatic erythematous multiple lesions on the back. On clinical examination, lesions were red and brown in color, well-defined within border, non-tender and no scales were present. There was not any significant family or medical history. No history of trauma, previous procedure or radiotherapy at the site of his vascular lesions,but the patientgave history of very frequent sunbaths. The lesions ranged in size from 4*4mm to 8*8mm and were not blanched under diascope pressure. Dermatoscopy of the lesions showed shiny white structures distributed throughout the lesions. There were no keratin features,pigmented clues or ulceration in the lesions.^7^ There was no obvious vasculature at the lesion site.

The preliminary diagnosis was basal cell carcinoma. The patient was undertaken the treatment with local surgical excision. On histopathological examination there was a band like proliferation of capillary blood vessels involving mostly the superficial dermis, and they were arranged parallel to the epidermis and confined to the upper reticular dermis. There was no cytologic atypia or increased mitotic activity in our case. The newly formed capillaries were surrounded by collagen bundles showing intense solar elastosis. There wasnotany inflammation. Immunohistochemical staining with CD31, D2-40, SMA and “FVIII” was performed and it was strongly positive for all of them which suggest, a specific marker for lymphatic differentiation.^8^ A peripheral ring of actinpositive (Alpha smooth muscle) pericytes were seen. EVG strain showed positivity in dermal stroma with irregularly thickened elastic fibers with disorganized arrangement.

**Figure 4:**
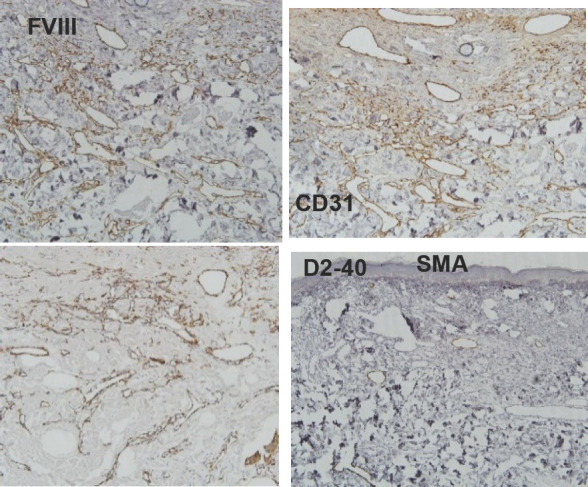
Immunohistopathology shows strong expression of CD31, FVIII D2-40 and SMA.

**Figure 5:**
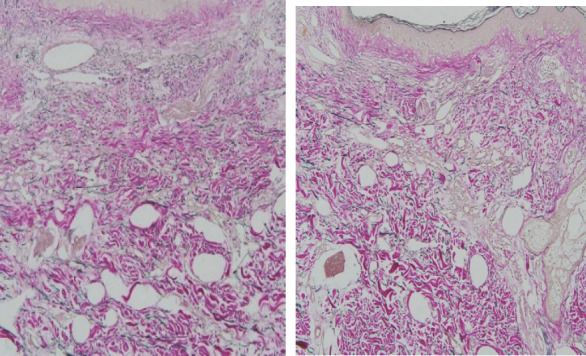
EVG stain showing irregularly thickened elastic fibers with disorganized arrangement.

## DISCUSSION

Acquired elastotic hemangioma is a benign vascular proliferation. It is a cutaneous hemangioma variant which is related with the sun exposure. AEH was first described by Requena et al in 2002 with a series of six cases.^1^ Characteristically, it presents as flat or raised, erythematous or violaceous, well-defined or irregular and slow growing, either solitary or multiple. It is usually asymptomatic. Most commonly located in the sun exposed sites such as forearms, neck and back; classically occurs in middle aged or elderly women but one study showed slight male predominace.^8^ In case of perimenopausal women, initiation of progesterone therapy might be the reason of multiple acquired elastotic hemangioma.

However, unlike other published cases, our patient's lesions were multiple but histopathology revealed the same characteristics as in other cases, confirming the diagnosis of AEH. The diagnosis is made on the basis of histopathological features and immunohistochemistry. AEH has a slow growth rate. Histologically, the most characteristics finding is a band like proliferation of the capillary blood vessels which are arranged parallel to the epidermis and are confined to the superficial dermis. The most classic feature is intense solar elastosis.^5^ The endothelial cells display a “hobnail” pattern without cellular atypia or mitosis. Immunohistochemistry shows the endothelial nature of the neoplastic cells which is usually positive for CD31, CD34 and D2-40 markers. The histopathologic differential diagnosis of AEH include acquired tufted hemangioma, hobnail hemangiomas, cherry angiomas, Mali's acroangiodermatitis and early Kaposi's sarcoma with angiomatous pattern. Acquired tufted hemangioma in histopathology shows a “cannonball” pattern with lobules of capillary tufts scattered in the dermis and subcutaneous fat. Similarly, kaposi's sarcoma histologically shows jagged vascular spaces lined by endothelial cells with a lymphoplasmacytic infiltrate, red blood cell extravasation and positive staining for HHV8.^6^

None of these entities show band like capillaries arranged along the superficial dermis with solar elastosis as characteristically seen in elastotic hemangioma. Clinical differential diagnosis of AEH according to literature include BCC, granuloma annulare, patch stage Kaposi sarcoma, acquired tufted angioma, targetoid hemosiderotic hemangioma, low grade angiosarcoma and capillary hemangioma.^4,5,9^ The dermatoscopy of AEH revealed prominent and widespread, shiny white structures distributed evenly throughout the lesion which is very specific. The etiopathology of acquired elastotic hemangioma is still not known, but the finding of solar elastosis supports the role of long-term sun exposure. In most cases there is no history of trauma. In our case the patient had history of frequent sun baths so the question arises of ultraviolet rays in developing acquired elastotic hemangioma. AEH is underdiagnosed and very often misdiagnosed as conventional hemangioma, thus, through this article we want to clarify the importance of this new hemangioma variant and enhance the possibility of this acquired elastotic hemangioma when clinicians encounter an older individual with a red plaque or multiple red lesions on the sun exposed site.

## Consent:

**JNMA Case Report Consent Form** was signed by the patient and the original article is attached with the patient's chart.

## Conflict of Interest

**None.**
